# Torsion of the Greater Omentum: A Mimicker of Acute Appendicitis

**DOI:** 10.7759/cureus.38165

**Published:** 2023-04-26

**Authors:** Ryosuke Yachi, Sho Fujiwara, Ryujiro Akaishi, Hiroshi Suzuki, Toru Hoshida

**Affiliations:** 1 Surgery, Iwate Prefectural Ofunato Hospital, Ofunato, JPN

**Keywords:** secondary omental torsion, omental torsion, laparoscopic surgery, mimicker of acute appendicitis, torsion of the greater omentum

## Abstract

Torsion of the greater omentum is rare and difficult to diagnose preoperatively. There are operative or non-operative treatment options. Operative management is often done for patients with abdominal pain in the right lower quadrant because omental torsion is misdiagnosed as appendicitis. If omental torsion is accurately diagnosed, previous reports suggest that symptoms may improve 12-120 hours after non-operative management of a primary omental torsion. Here, we report a successful case of surgical treatment for torsion of the greater omentum after non-operative treatment was ineffective. Thus, considering the severity of the pain and operative risk, a laparoscopic omentectomy may be feasible to relieve the severe abdominal pain promptly.

## Introduction

Torsion of the greater omentum is a rare disease, and it occurs in <0.4% of the population [1]. It can occur spontaneously or can be caused by events such as abdominal surgery, herniation, trauma, and inflammation. Especially when the omental torsion occurs in the right lower quadrant, it is difficult to diagnose from physical examination alone because the symptoms mimic those of appendicitis. Furthermore, treatment options have not been established. Here, we report a successful case of surgical treatment for torsion of the greater omentum after non-operative treatment was not effective.

## Case presentation

**Figure 1:**
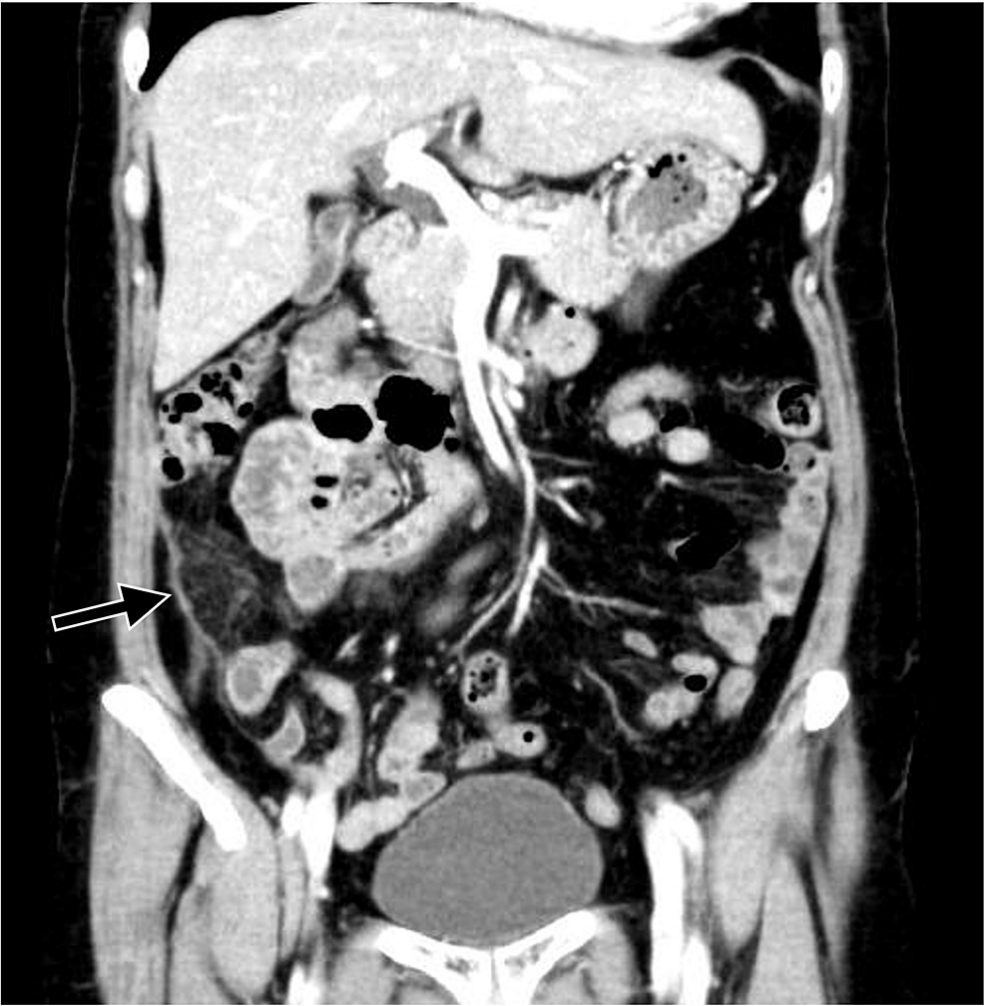
Preoperative contrast-enhanced computed tomography. Contrast-enhanced computed tomography shows that the greater omentum adjacent to the appendix is locally hyperdense (arrow).

A 59-year-old woman with a one-day history of acute abdominal pain in the right lower quadrant and fever presented to the hospital. The right lower abdominal quadrant exhibited localized rebound tenderness and muscular guarding on physical examination. The most likely diagnosis was acute appendicitis. A computed tomography (CT) investigation revealed that the appendiceal wall was mildly thickened, which was not enough to diagnose appendicitis definitively. However, it did show that the greater omentum adjacent to the appendix displayed locally hyperdense fat, which was not consistent with peri-appendiceal inflammatory changes seen in appendicitis (Figure 1).

CT did not show enough inflammation in the appendix to cause localized inflammation of the greater omentum, suggesting the possibility of torsion or infarction of the greater omentum. We administered intravenous cefmetazole 1 g every eight hours empirically and acetaminophen 1 g every six hours for two days, but this did not relieve the patient’s severe abdominal pain.

**Figure 2:**
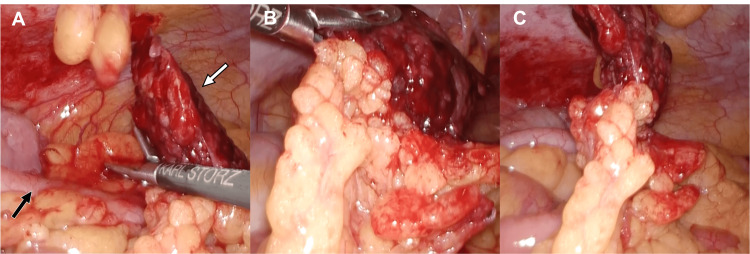
Intraoperative finding. The intraoperative finding reveals the locally necrotic omentum with torsion (white arrow) and intact appendix (black arrow).

The patient underwent laparoscopic omentectomy and appendectomy because her abdominal pain did not improve. Intraoperative findings revealed that the ischemic omentum adhered to the appendix and abdominal wall with inflammation (Figure 2). The histopathology of the resected omentum revealed congestion, necrosis, and hemorrhage in fat tissue. Her abdominal pain was relieved after surgery, and she was discharged on postoperative day three without any complications.

## Discussion

We noted two important clinical issues. Torsion of the right side greater omentum is challenging to diagnose from a physical examination. CT is essential to diagnose omental torsion and rule out appendicitis when severe pain, laboratory data, and vital signs are not enough to explain appendicitis. Furthermore, even if omental torsion can be treated by non-operative treatment, laparoscopic omentectomy is feasible when abdominal pain is severe or uncontrollable with enough analgesia.

First, torsion of the right side greater omentum is almost impossible to diagnose from only a physical examination. Omental torsion, classified as a primary or secondary torsion, is rare [1]. It is not easy to diagnose from a physical examination. Even on CT, it is not easy to diagnose preoperatively [2]. A previous report revealed that <5% of all cases are accurately diagnosed preoperatively [1]. In our case, physical examination suggested appendicitis, but fever and inflammation of laboratory data were not enough to explain appendicitis. Finally, CT was required to diagnose omental torsion. We must consider CT and omental torsion when data is inconsistent with appendicitis but severe abdominal pain.

Moreover, laparoscopic omentectomy is feasible when abdominal pain is severe or uncontrollable with enough analgesia. In fact, there are operative or non-operative treatment options [3,4]. A previous study reported that although torsion of the omentum was treatable non-operatively, symptoms may improve 12-120 hours after non-operative management of a primary omental torsion [3]. Furthermore, in most cases, it can take more than three days [3]. In our case, severe abdominal pain continued for two days with enough analgesia without fever. Another previous report suggested that a laparoscopic omentectomy should be done considering the severity of pain and risk [4]. Thus, we performed a laparoscopic omentectomy to relieve the patient’s uncontrollable pain.

In conclusion, omental torsion is rare, but CT is essential to diagnose omental torsion. Laparoscopic omentectomy is feasible when abdominal pain is severe or uncontrollable with enough analgesia considering the operative risk. Further studies should be done to determine the actual clinical courses and operative risk because only fewer than 5% of cases are diagnosed preoperatively.

## Conclusions

Although omental torsion may be treated non-operatively, improving patients’ symptoms could often take a long time. Most cases of laparoscopic surgery for omental torsion do not require complicated procedures. Thus, laparoscopic surgery may be feasible considering the severity of the pain and patients’ risk.
